# Steam explosion pretreatment of softwood: the effect of the explosive decompression on enzymatic digestibility

**DOI:** 10.1186/s13068-016-0567-1

**Published:** 2016-07-22

**Authors:** Thomas Pielhop, Janick Amgarten, Philipp Rudolf von Rohr, Michael H. Studer

**Affiliations:** Transport Processes and Reactions Laboratory, Institute of Process Engineering, ETH Zurich, Sonneggstrasse 3, 8092 Zurich, Switzerland; Laboratory for Bioenergy and Biochemicals, School of Agricultural, Forest and Food Sciences, Bern University of Applied Sciences, Länggasse 85, 3052 Zollikofen, Switzerland

**Keywords:** Steam explosion, Pretreatment, Lignocellulosic biomass, Softwood, Enzymatic digestibility, Biorefinery

## Abstract

**Background:**

Steam explosion pretreatment has been examined in many studies for enhancing the enzymatic digestibility of lignocellulosic biomass and is currently the most common pretreatment method in commercial biorefineries. The information available about the effect of the explosive decompression on the biochemical conversion is, however, very limited, and no studies prove that the latter is actually enhanced by the explosion. Hence, it is of great value to discern between the effect of the explosion on the one hand and the steaming on the other hand, to identify their particular influences on enzymatic digestibility.

**Results:**

The effect of the explosive decompression in the steam explosion pretreatment of spruce wood chips on their enzymatic cellulose digestibility was studied systematically. The explosion had a high influence on digestibility, improving it by up to 90 % compared to a steam pretreatment without explosion. Two factors were identified to be essentially responsible for the effect of the explosion on enzymatic digestibility: pretreatment severity and pressure difference of the explosion. A higher pretreatment severity can soften up and weaken the lignocellulose structure more, so that the explosion can better break up the biomass and decrease its particle size, which enhances its digestibility. In particular, increasing the pressure difference of the explosion leads to more defibration, a smaller particle size and a better digestibility. Though differences were found in the micro- and nanostructure of exploded and non-exploded biomass, the only influence of the explosion on digestibility was found to be the macroscopic particle size reduction. Steam explosion treatments with a high severity and a high pressure difference of the explosion lead to a comparatively high cellulose digestibility of the—typically very recalcitrant—softwood biomass.

**Conclusions:**

This is the first study to show that explosion can enhance the enzymatic digestibility of lignocellulosic biomass. If the enhancing effect of the explosion is thoroughly exploited, even very recalcitrant biomass like softwood can be made enzymatically digestible.

**Electronic supplementary material:**

The online version of this article (doi:10.1186/s13068-016-0567-1) contains supplementary material, which is available to authorized users.

## Background

The pretreatment of lignocellulosic biomass for enhancing the enzymatic bioconversion of the carbohydrates (cellulose and hemicellulose) to their sugars is an intensively studied subject. Steam explosion as a pretreatment method is one of the most efficient and most commonly used. Advantages of steam explosion pretreatment include low capital investment, moderate energy requirements and low environmental impacts [1–3]. No acid, base or solvent chemicals are required which simplifies the subsequent biorefinery stages and reduces their cost: lower detoxification effort due to less formation of compounds inhibiting enzymatic hydrolysis and fermentation [4]; minimized need for neutralization chemicals [5] and no need for the removal of an organic lignin solvent, which can be inhibitory to cellulase enzymes and fermentative microorganisms [4]. A steam gun is also well suited to deal with large biomass particles reducing their size during the explosion step in an energy-efficient way [6–8], and the process is related to sulfite pulping, a technically mature large-scale process. For all these reasons, continuous steam explosion pretreatments are also dominating on a commercial scale [8].

It is a flexible technology which has proven effective for a great variety of lignocellulosic feedstock, including hardwoods, grasses and agricultural residues such as corn stover, sugarcane bagasse and wheat straw [2, 4, 9, 10]. However, it is much less effective for softwood [11, 12], which is considered to be one of the worst-case scenarios as a feedstock for bioconversion processes due to its high lignin content and recalcitrance [2]. Therefore, most planned or operating commercial-scale biorefineries based on enzymatic bioconversion use a different feedstock [13], and currently, only one project that will use softwood is planned in Kajaani, Finland. A review on pretreatment methods for softwood describes acid-catalyzed steam pretreatment—e.g. using H_2_SO_4_ or gaseous SO_2_—as the most suitable pretreatment method for softwood [14]. However, chemical usage is associated with additional costs for neutralization as well as recycling [11], and SO_2_ is highly toxic and may present negative safety and environmental impacts [12, 14, 15]. Reducing softwood recalcitrance via (uncatalyzed) cost-effective steam pretreatment methods would, therefore, be of great benefit.

In steam explosion pretreatments, the biomass is first treated with saturated steam for a certain amount of time. The accessibility of steam into the inner structures of lignocellulosics is high due to the high vapor-phase diffusion [16], and it has been reported that the maximum of adsorbed water in e.g., wood chips is reached after 100 s [17]. The biomass is heated up by the condensation of steam so that its “capillary-like” microporous structure is soaked with liquid hot water [16]. This causes the release of acids from the hemicellulose fraction, lowering the pH to 3–4 [5, 18]. The moderate acidic conditions can especially hydrolyze the hemicellulose (autohydrolysis), but cleavage of lignin ether bonds is also included [19–21]. This breaking down of the lignocellulose structure and the removal of hemicellulose improve the enzymatic digestibility. At the end of the steam treatment, the pressure is released abruptly, for example, by opening a reactor valve which discharges the biomass slurry into a blow tank (“steam gun”). Due to the sudden drop of pressure—normally to environmental pressure of 1 bar—flash evaporation of superheated water occurs. The sudden evaporation of the “inner water” forces, literally, an explosion of the biomass and causes extensive defibration and even defibrillation and rupture of fibers [16].

Though steam explosion pretreatment for the enzymatic hydrolysis of lignocellulosic biomass has been intensively studied for decades, very little information is available regarding the effect of the explosion itself on enzymatic digestibility. Moreover, the few opinions that can be ascertained from the scientific community and the literature are controversial and inconsistent. On the one hand, it has been stated that the explosion in a pretreatment for bioconversion offers the advantage of efficiently decreasing particle size [1, 22], disrupting plant cells [10] and making the biomass more bouffant, resulting in an increased surface area and better accessibility of carbohydrates to enzymes [23, 24]. However, no study has been conducted to verify if those observations do really have an influence on enzymatic digestibility, and no direct proof for those conclusions has been presented in the literature so far. On the contrary, it has been reported that the high level of digestibility provided by steam explosion is primarily due to chemical autohydrolysis reactions and not due to the mechanical disruption during explosive decompression [11]. This has been concluded on the basis of—to the best of our knowledge—the only available systematic experimental study on this subject: Brownell et al. [25] demonstrated that aspen wood chips which underwent steam treatment with gentle decompression were as easy to hydrolyze as chips that underwent steam treatment and explosive decompression. The treatment with saturated steam at 240 °C gave essentially the same yields of glucose and total reducing sugars, whether or not 80 % of the steam was bled off before explosion and even if the chips remained intact [25]. It was, therefore, concluded that the explosion is—at least in the case of aspen wood—unnecessary for the development of cellulose accessibility [25]. These findings were supported by similar studies with aspen wood [26, 27], and it was even questioned whether the explosion serves any purpose other than to empty the steam gun [26]. It has been acknowledged that the explosion opens up the particulate structure of the biomass, but cellulose digestibility is supposed to hardly correlate with this physical effect [5].

In summary, the only proven positive effect of the explosion for a biorefinery concept is that it decreases the particle size—in that way, enhancing the pumpability and handling of the biomass slurry. This article aims at further closing the knowledge gap of the explosion’s effect on enzymatic digestibility. Can it have an effect—and if so—what characteristics of the biomass are affected that enhance its digestibility and how can this be intensified? For this purpose, several steam explosion studies were conducted with softwood as a feedstock. Due to its high recalcitrance to bioconversion, it may be easier to reveal positive effects of the explosion.

## Methods

### Biomass

Spruce wood chips were prepared from a roughly 30-year-old tree, cut in summer 2014 in Biberist (canton of Solothurn, Switzerland) by chopping through a 30-mm screen. The fresh biomass had a dry matter of 46.2 ± 1.7 %, and the composition was determined to be: glucan 39.6 ± 0.9 %, mannan 17.7 ± 1.6 %, acid-soluble lignin (ASL) 5.22 ± 0.04 %, acid-insoluble lignin (AIL) 29.0 ± 0.2 % and extractives 6.6 ± 0.4 % (total 98.1 %). After chopping, the wood chips were stored at 5 °C in sealed plastic bags during the experimental time of 4 months.

### Steam gun

**Fig. 1:**
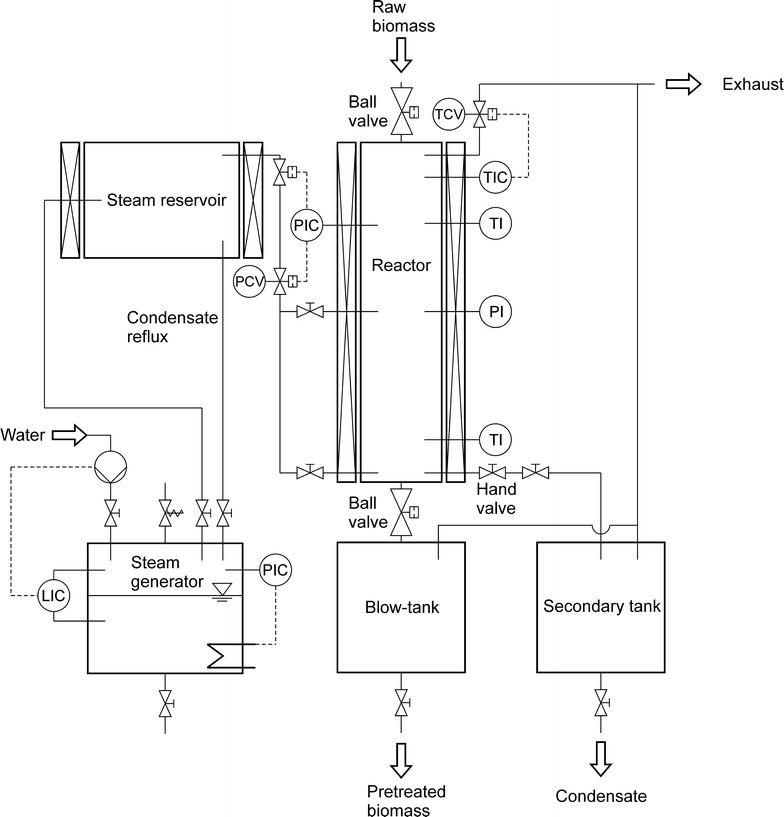
Schematic of the steam explosion system (“steam gun”)

The steam gun was set up by the Industrieanlagen Planungsgesellschaft (IAP; Graz, Austria). A schematic of the steam explosion system is shown in Fig. 1. Steam of 32 bar is generated in a boiler (EP18 Electropack; Fulton, Great Britain) with a power of 18 kW. An additional steam reservoir between boiler and reactor gives further storage capacity for 23 l of steam. The pretreatment reactor is made of stainless steel (type 1.4571) with a volume of 5.8 l (DN100 i.e. 114.3 mm inner Ø, 700 mm inner height) and can be loaded with biomass via a pneumatic ball valve on top. Saturated steam can be injected into the reactor both at the bottom and at half height of the reactor. Steam injection is controlled by the pressure setpoint in the reactor. Entrapped air has to be removed to reach the steam saturation temperature corresponding to the pressure setpoint. Therefore, an exhaust valve at the top of the reactor allows for deaeration once that steam is injected. The exhaust valve is controlled by the temperature difference measured at the top of the reactor—where air accumulates due to its lower density—and the temperature corresponding to saturated steam. A ball valve (Ballostar KHA DN100; Klinger, Germany) at the bottom of the reactor allows for the discharge of the biomass into the blow tank. The valve is driven by a pneumatic actuator operated at 10 bar air pressure to ensure a fast opening and depressurization as needed for steam explosion. Alternatively, pressure can also be released via a hand valve at the bottom of the reactor into a secondary tank.

### Pretreatment

The boiler was heated to provide steam of 32 bar. Pretreatment experiments with a steam pressure in the reactor of 11, 19 and 31 bar (absolute pressure) were carried out, corresponding to temperatures of 184, 210 and 235 °C. Before each experiment, the reactor was first pre-heated by allowing it to stand filled with steam of the desired experimental temperature for 10 min to reduce steam condensation during the actual experiment and the time required to bring the metal reactor to the desired temperature. The steam and condensate were released again, and the reactor was immediately loaded with 1.5 kg of fresh spruce wood chips and repressurized. Steam was injected only at the bottom of the reactor to whirl the biomass and ensure good contact with steam. Time zero for the treatment was taken when the pressure in the reactor reached 99 % (experiments at 11 and 19 bar) or 95 % (experiments at 31 bar) of the target pressure. This translates to starting the time measurement 50–60 s after beginning of the steam injection. Entrapped air was removed within the first 3 min so that the saturated steam temperature was reached. After a defined pretreatment time, the biomass was exploded/discharged into the blow tank. The obtained slurry was weighed and then filtered recording weight and pH of the filtrate. The biomass filter cake was not washed, and its dry matter content was determined in duplicate. In experiments to study the influence of different explosion pressures, the pressure was partially released shortly before opening the bottom ball valve at the end of pretreatment. Therefore, the steam inlet valve was closed, and pressure was bled off via the hand valve into the secondary tank. The valve was opened only minimally so that only steam/condensate but no biomass solids could escape. The adjustment to a certain pressure took about 20 s before the biomass was then discharged with a reduced pressure into the blow tank. A minimum overpressure of 2.5 bar was necessary for emptying the gun, and experiments which were conducted with this low explosion pressure are referred to as experiments “without explosion” in this work. The biomass slurry recovered from the blow tank was filtered and the biomass processed as described above. The filtrate was mixed with the liquid recovered from the secondary tank to record weight and pH of the mix.

Several experiments were conducted with varying the pretreatment temperature, time and pressure difference of the explosion. An overview of the experimental conditions and pretreatment severities is shown in Table 1. The pretreatment severity was defined as Eq. (1):

1 $$R_{0} = t \cdot {\text{e}}^{{\frac{T - 100}{14.75}}} \;,\;$$

where *t* is the pretreatment time in minutes and *T* the pretreatment temperature in degrees Celsius [28].

**Table 1:** Overview of pretreatment experiments and experimental conditions

*T*/°C	*p*_abs_/bar	*t*/min	log *R*_0_/–	Δ*p* explosion/bar
184	11	5	3.2	10
184	11	5	3.2	2.5^a^
210	19	5	3.9	18
210	19	5	3.9	2.5^a^
235	31	2.5	4.4	30
235	31	5	4.7	30
235	31	10	5.0	30
235	31	15	5.2	30
235	31	20	5.3	30
235	31	2.5	4.4	2.5^a^
235	31	5	4.7	2.5^a^
235	31	10	5.0	2.5^a^
235	31	15	5.2	2.5^a^
235	31	20	5.3	2.5^a^
235	31	10	5.0	5
235	31	10	5.0	7.5
235	31	10	5.0	10
235	31	10	5.0	15
235	31	10	5.0	20
235	31	10	5.0	25

^a^Experiments with a Δ*p* of 2.5 bar are referred to as experiments “without explosion”

### Biomass analysis

The dry matter and composition (glucan, mannan, AIL, ASL, extractives) of the raw and pretreated biomass as well as the sugar contents (glucose, sum of hemicellulosic sugars) in the pretreatment liquors were determined by the methods published by the National Renewable Energy Laboratory (NREL) [29–33]. The dried pretreated biomass was, however, pulverized by pestling before compositional analysis. All biomass and pretreatment liquor analyses were done in triplicate and duplicate, respectively, and single standard deviations are reported with the mean in this work.

### Enzymatic hydrolysis

The pretreated biomass recovered by filtration underwent enzymatic hydrolysis according to the NREL standard procedure [34] with a cellulose concentration of 1 %w/w. The following changes were made: sodium azide at a final concentration of 0.01 g l^−1^ was used instead of antibiotics, and the pH was adjusted to 5.0 (0.05 mol l^−1^ sodium citrate buffer after sample preparation). 10-ml samples were prepared in 20-ml scintillation vials (VWR, USA). Due to the larger particle size, raw biomass or biomass that had been pretreated with a pressure difference lower than 15 bar in the explosion was prepared as 150-ml samples in 250-ml laboratory glass bottles (Schott, Germany). Accellerase 1500 (Genencor; lot number 4901298419), with an activity of 26 filter paper units (FPU) ml^−1^ measured according to Adney and Baker [35], was used with final concentrations of 15, 30 or 60 FPU g^−1^ cellulose in the sample preparation. Samples were incubated in a shaker (Multitron; Infors-HT) with a shaking throw of 25 mm at 50 °C and 210 rpm for 120 h, and then analyzed for sugars in the supernatant. All hydrolysis experiments were carried out in triplicate, and single standard deviations are reported with the mean.

### Sugar analysis

Sugar analysis by HPLC (high-performance liquid chromatography) was performed as described by Sluiter et al. [30] using a Waters 2695 separation module equipped with a Waters 410 differential refractometer and a Bio-Rad Aminex HPX-87H column.

### Yield calculations

The enzymatic cellulose digestibility was calculated as defined below.

2 $${\text{Digestibility}}_{\text{Cellulose}} = \frac{{m_{{{\text{Glucose,EH }} {\text{sample}}}} \cdot 0.9}}{{m_{{{\text{Glucan}}, {\text{EH }} {\text{sample}}}} }}\;,$$

where *m*_Glucose,EH sample_ is the mass of glucose released during the enzymatic hydrolysis experiment, and *m*_Glucan,EH sample_ is the mass of glucan added to the experiment with the biomass sample. The factor 0.9 accounts for the conversion of the anhydrous polymer to the monosaccharide. The hemicellulose (mannan) digestibility was calculated analogously.

The corresponding theoretical glucose yield that can be obtained from the recovered pretreated biomass was calculated as defined in the following equation.

3 $${\text{Yield}}_{{{\text{Glucose}},{\text{EH}}}} = {\text{Digestibility}}_{\text{Cellulose}} \cdot \frac{{m_{{{\text{Glucan}},{\text{Recovered}}}} }}{{m_{{{\text{Glucan}},{\text{Feedstock}}}} }}\;,$$

where *m*_Glucan,Recovered_ is the mass of glucan recovered with the pretreated biomass, and *m*_Glucan,Feedstock_ is the mass of glucan added to pretreatment with the feedstock. In that way, losses due to glucan degradation in pretreatment and re-collecting of the pretreated biomass are accounted for. The enzymatic hydrolysis yield from hemicellulose was calculated analogously.

The yield of glucose released to the pretreatment liquor was calculated as defined in the following equation.

4 $${\text{Yield}}_{{{\text{Glucose}},{\text{Pretreatment }} {\text{liquor}}}} = \frac{{m_{{{\text{Glucose}},{\text{Pretreatment }} {\text{liquor}}}} \cdot 0.9}}{{m_{{{\text{Glucan}},{\text{Feedstock}}}} }}\;,$$

where *m*_Glucose,Pretreatment liquor_ is the mass of glucose in the recovered pretreatment liquor. The yield of hemicellulosic sugars (represented as mannose) in the pretreatment liquor was calculated analogously.

The total glucose yield summing up the glucose yields from pretreatment and enzymatic hydrolysis was calculated as defined in the following equation.

5 $${\text{Yield}}_{{{\text{Glucose}},{\text{Total}}}} = {\text{Yield}}_{{{\text{Glucose}},{\text{Pretreatment }} {\text{liquor}}}} + {\text{Yield}}_{{{\text{Glucose}},{\text{EH}}}}$$

The total yield of hemicellulosic sugars (represented as mannose) was calculated analogously.

The total sugar yield summing up the glucose and hemicellulosic sugar yields from pretreatment and enzymatic hydrolysis was calculated as defined below.

6 $${\text{Yield}}_{{{\text{Sugar}},{\text{Total}}}} = \frac{{{\text{Yield}}_{{{\text{Glucose}},{\text{Total}}}} \cdot X_{{{\text{Glucan}},{\text{Feedstock}}}} + {\text{Yield}}_{{{\text{Mannose}},{\text{Total}}}} \cdot X_{{{\text{Mannan}},{\text{Feedstock}}}} }}{{X_{{{\text{Glucan}},{\text{Feedstock}}}} + X_{{{\text{Mannan}},{\text{Feedstock}}}} }}\;,$$

where *X*_Glucan,Feedstock_ and *X*_Mannan,Feedstock_ are the contents of glucan and mannan in the raw biomass on a dry matter basis.

### Sieving

The particle-size distribution of raw biomass and of the biomass pretreated with steam of 31 bar was analyzed by wet sieve analysis with a vibratory sieve shaker (AS200, Retsch). Sieving was carried out for 10 min with a water flow rate of 1 l min^−1^. The biomass samples contained at least 150 g of dry matter and were fractioned with mesh sizes of 6.7, 4, 2, 1, 0.5, 0.25, 0.18 and 0.025 mm and a sieving amplitude of 3 mm. The fraction passing the 0.025-mm sieve was sieved further with mesh sizes of 20, 16 and 10 μm under equal conditions, other than a sieving amplitude of 2 mm. The sieves with the biomass residues were dried overnight at 105 °C, and the weight difference to the empty sieves was recorded.

Sieving was also used to study the influence of particle size reduction by the explosion on enzymatic hydrolysis. To this end, eight fractions of the biomass pretreated with a severity of log *R*_0_ = 5.0 (*T* = 235 °C, *t* = 10 min) each with and without explosion (Δ*p* = 30 bar) were prepared, using mesh sizes of 6, 4, 2.8, 1.4, 1, 0.5, 0.355 and 0.25 mm with a sieving amplitude of 3 mm. Samples from the obtained sieving fractions were then taken for moisture analysis before preparing 10-ml samples for enzymatic hydrolysis as described above.

### Scanning electron microscopy

Selected biomass samples were studied by scanning electron microscopy (SEM). A small amount of freeze-dried (Flexi-dry, FTS systems, USA) biomass was fixed on conductive polycarbonate stickers with admixed graphite (G3347, Plano, Germany), and the samples were coated with a 3-nm-thick platinum layer in a sputter coater (MED 010, Bal-Tec, Liechtenstein). The electron microscope (Gemini1530, Zeiss, Germany) was operated at an acceleration voltage of 2 kV using a secondary electron detector [36].

## Results and discussion

### Influence of pretreatment severity on the explosion effect

The biomass loading in the reactor (dry weight of raw wood chips loaded to the reactor vs. weight of the slurry recovered after pretreatment) varied between 12 and 32 %w/w depending on the pretreatment time and temperature, showing the high loadings that are possible in steam explosion.

Low-severity pretreatments were carried out with pretreating the biomass for 5 min at 184 and 210 °C (log *R*_0_ = 3.2 and 3.9). Pretreatment at 184 °C with and without explosion lead to cellulose digestibilities of 10.9 ± 1.1 and 12.1 ± 0.9 %, respectively, of the pretreated biomass. Pretreatment at 210 °C with and without explosion lead to digestibilities of 24.0 ± 1.4 and 23.2 ± 0.9 %, respectively. Thus, the explosion did not have an influence on digestibility, and yields in hydrolysis were low. It did neither have a visible influence on the biomass particle size. The pretreatment severity was presumably not high enough to soften up and weaken the lignocellulose structure sufficiently, so that the explosion could not defibrate the wood chips. The applied explosion pressure differences of 10 and 18 bar can actually achieve a good defibration if pretreatment severity is higher (compare the following subchapter).

**Fig. 2:**
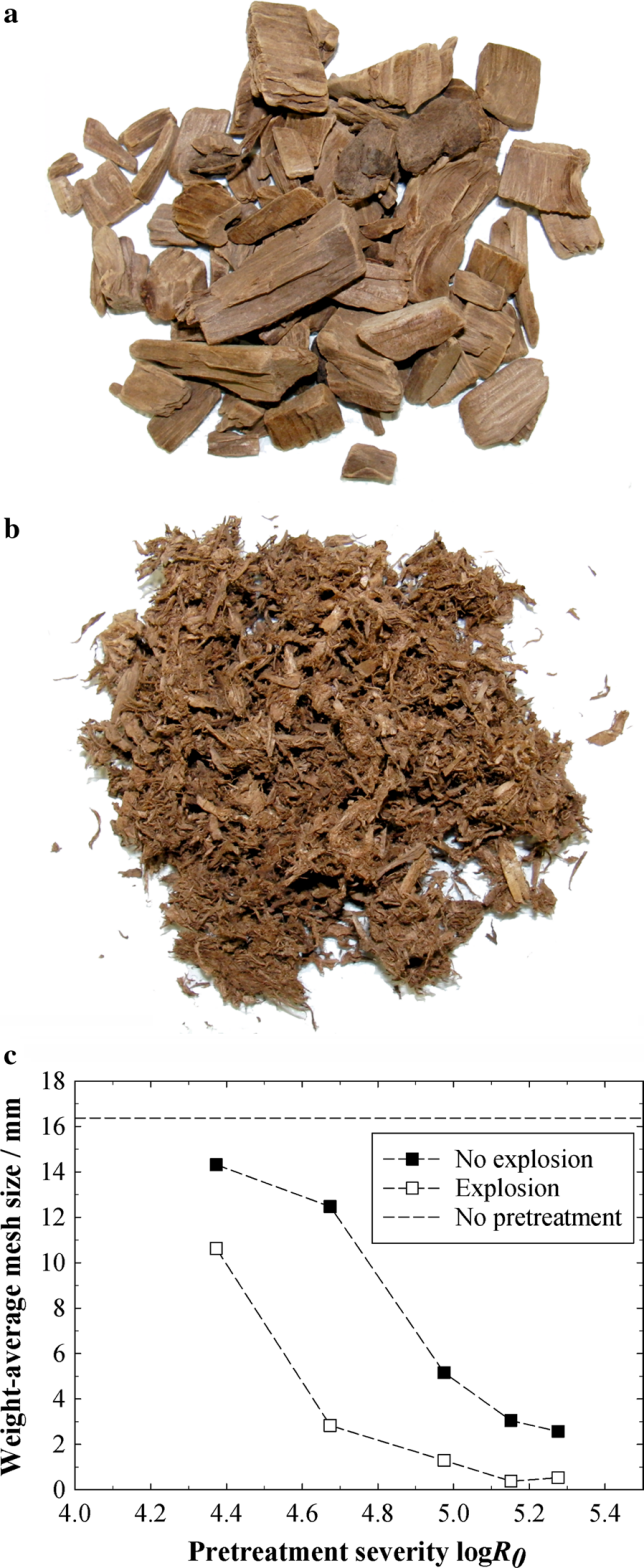
Steam explosion effect on biomass structure. Spruce wood chips after a steam pretreatment without (**a**) and with explosion (**b**). Shown are the dried screenings of the 6.7-mm sieve. Pretreatment conditions: log *R*_0_ = 4.4 (*T* = 235 °C, *t* = 2.5 min), Δ*p* explosion = 30 bar. **c** Weight-average particle size of spruce wood chips after steam pretreatments at different severities with and without explosion. Pretreatment conditions: *T* = 235 °C, *t* = 2.5–20 min, Δ*p* explosion = 30 bar

The explosion after steam pretreatments at 235 °C and a corresponding explosion pressure difference of 30 bar lead to a visible defibration (Fig. 2b), with the highest severities even allowing for a pulverization of the wood chips. In contrary, wood chips in pretreatments without explosion largely remained intact (Fig. 2a). The weight-average particle size (here: weight-average mesh size of the sieving analysis) of the pretreated biomass could be decreased by the explosion in every experiment (Fig. 2c). Increasing the severity in the experiments with explosive discharge lead to smaller particles, as the lignocellulosic structure was broken up more by the harsher treatment, and the explosion could have a higher fragmenting effect. For very high severities >5.1, it seems that the size-reducing effect of the explosion stagnates, and the average size cannot be reduced to less than around 0.4-mm mesh size. Interestingly, increasing severity also lead to a larger reduction in the particle size of the biomass pretreated without explosion. It has to be noted, however, that the wet sieving procedure itself might also induce a certain reduction of particle size, since the wood chips became “crumbly” by the pretreatment. An exemplary particle-size distribution of raw, exploded and non-exploded biomass is shown in Additional file 1: Fig. S1.

No major differences can be observed in the compositional analysis of exploded and non-exploded biomass (Additional file 1: Fig. S2). Hemicellulose contents are low (2–11 %), since it is dissolved fast due to autohydrolysis. Similar to the compositional analysis of the biomass, no differences can be found in the pH of the recovered pretreatment liquors (Additional file 1: Fig. S3). It decreases with increasing severity from around 3.4 to 3.15 because of the release of acids from the biomass. Generally, pH was distinctly lower compared to the low-severity pretreatments at 184 and 210 °C (pH 4.25–3.9, also shown in Additional file 1: Fig. S3), which certainly enhanced the pretreatment effect by catalyzing the decomposition of the lignocellulose structure.

**Fig. 3:**
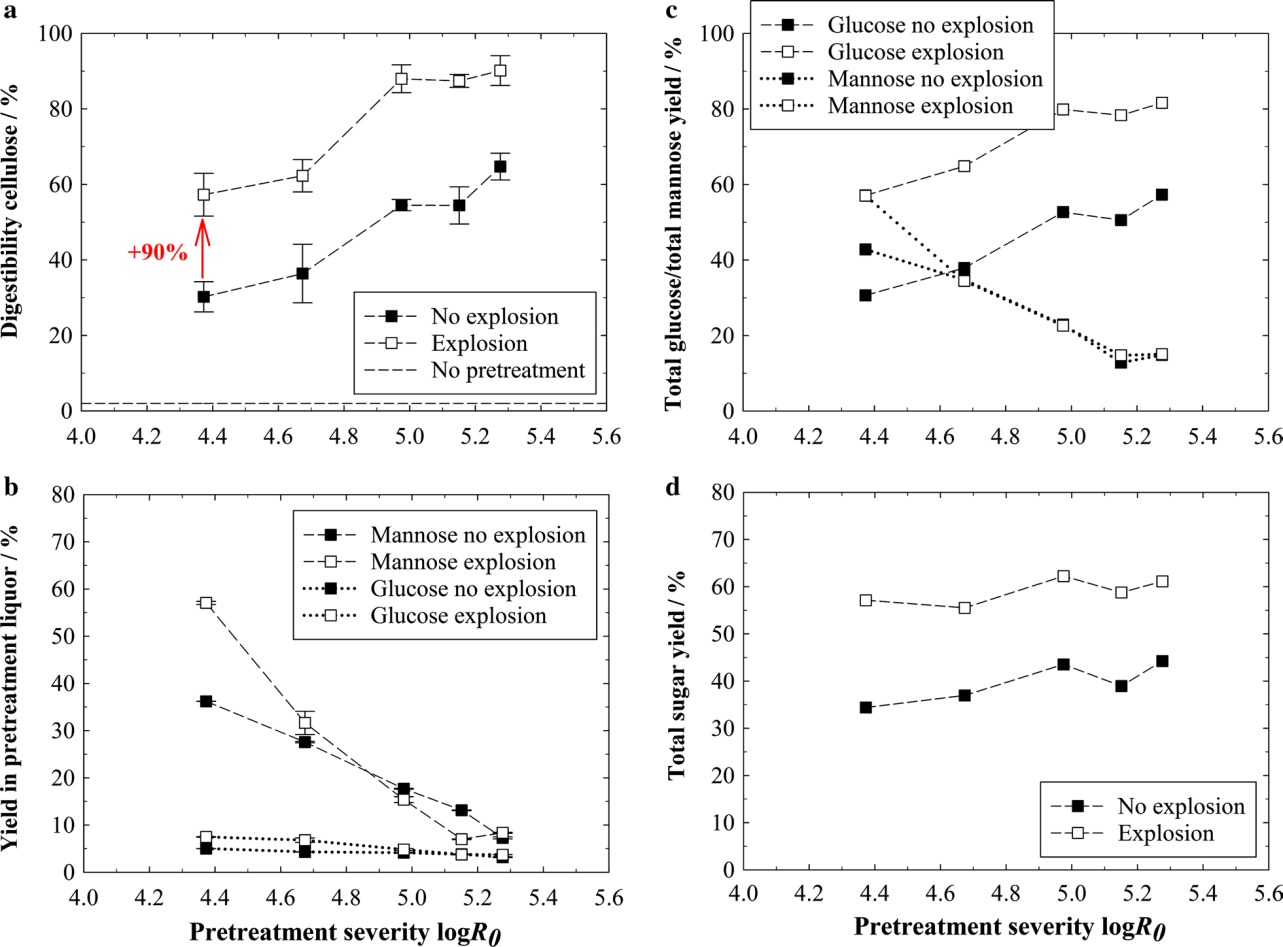
Sugar yields after steam pretreatments of spruce wood chips at different severities with and without explosion. **a** Enzymatic cellulose digestibility. Cellulose conversion to glucose is expressed as % of pretreated biomass content. **b** Yields of glucose and mannose dissolved in the pretreatment liquor, expressed as % of raw biomass content. **c** Total glucose and mannose yields from the combined operations of pretreatment and enzymatic hydrolysis, expressed as % of raw biomass content. **d** Total sugar yield of glucose plus mannose from the combined operations of pretreatment and enzymatic hydrolysis, expressed as % of raw biomass content. Pretreatment conditions: *T* = 235 °C, *t* = 2.5–20 min, Δ*p* explosion = 30 bar; hydrolysis conditions: 1 %w/w cellulose, 60 FPU g^−1^ cellulose

The corresponding cellulose digestibilities in enzymatic hydrolysis are shown in Fig. 3a. The hydrolyzability of raw wood chips is also represented, showing that they are practically not digestible. Pretreatments without explosion enhance the digestibility up to 65 %. It increases with severity as more hemicellulose gets extracted (Additional file 1: Fig. S2a) and the lignocellulosic structure is broken up more. Subjecting the biomass to an explosion after the pretreatment gives a high and continuous additional benefit, increasing the digestibility in every experiment and by up to 90 % relative to the pretreatments without explosion.

The results demonstrate that the explosion in steam explosion pretreatments can actually have a high effect in enhancing the enzymatic digestibility of lignocellulosic biomass. Moreover, it can render a comparatively well hydrolyzable cellulose fraction from softwood—a biomass originally thought to be too recalcitrant for such a pretreatment not using any chemicals. The review by Galbe and Zacchi [14] compares several one- and two-stage acid-catalyzed steam explosion pretreatment methods for softwood, using enzyme dosages of 30–60 FPU g^−1^ cellulose in hydrolysis similar to the work at hand (60 FPU g^−1^ cellulose). The presented total glucose yields—yields added up from enzymatic hydrolysis and the glucose dissolved in the pretreatment liquor—range between 52 and 80 %. The not-yet optimized steam explosion pretreatment of the present work allowed for total glucose yields of even 82 % (compare Fig. 3c). This points out that if the beneficial effect of the explosion is exploited, steam explosion can, indeed, be an effective pretreatment method for softwood.

The yields of glucose and mannose in the pretreatment liquor are shown in Fig. 3b. The highest mannose yield of 57 % is reached at the lowest severity. Mannose yields decline considerably with increasing severity, since dissolved hexoses degrade to e.g., 5-hydroxymethylfurfural (HMF) during steam pretreatment [4, 5]. Interestingly, mannose yields in the pretreatment liquor seem to be increased in experiments with explosion at lower severities. The same holds true for the glucose yields in the liquor. The reasons for this observation have still to be elucidated, though. The explosion did not influence the enzymatic hemicellulose digestibility (results not shown). As most of the hemicellulose got already dissolved in pretreatment, the total mannose yields (Fig. 3c) were similar to the mannose yield in the pretreatment liquor. The highest total sugar yield of 62 %—summing up all glucose and mannose yields from the pretreatment liquor and hydrolysis—is reached with explosion at a severity of 4.7 (Fig. 3d).

The total sugar yields are limited due to the degradation of hemicellulosic sugars in pretreatment. In an actual process, it is certainly desired to enhance sugar yields by a two-stage pretreatment, which is also carried out commercially [8]. A first mild pretreatment stage recovers hemicellulosic sugars in high yields, and a harsher second stage enhances the cellulose digestibility. This can also reduce the formation of degradation products from hemicellulosic sugars, such as furfural or HMF, which are inhibitory to microbial fermentation [5].

### Influence of pressure difference on the explosion effect

**Fig. 4:**
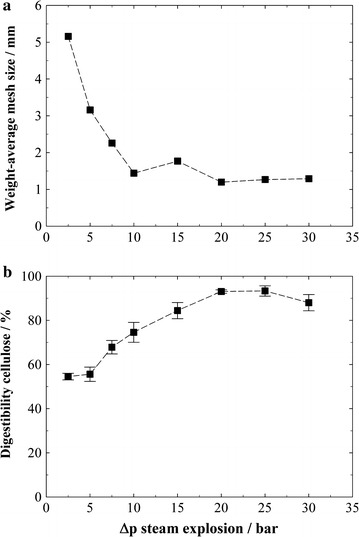
Effect of the pressure difference in explosive decompression. **a** Weight-average particle size of spruce wood chips after steam explosion pretreatments. **b** Cellulose digestibility after steam explosion pretreatments. Cellulose conversion to glucose is expressed as % of pretreated biomass content. Pretreatment conditions: log *R*_0_ = 5.0 (*T* = 235 °C, *t* = 10 min); hydrolysis conditions: 1 %w/w cellulose, 60 FPU g^−1^ cellulose

Since the explosion had a high influence on biomass digestibility, the dependence of digestibility on the pressure difference Δ*p* leading to the explosion was studied. Therefore, several pretreatments were carried out at a constant severity, where the explosion had led to a high improvement and a high digestibility (*T* = 235 °C, *t* = 10 min, log *R*_0_ = 5.0; compare Fig. 3a). The explosion pressure was varied/reduced by bleeding off a part of the steam (and condensate) via the hand valve shortly before the explosion. It was observed that a minimal Δ*p* > 5 bar is needed to actually see an effect of the explosion on digestibility, and increasing Δp further considerably enhances it (Fig. 4b). The higher pressure difference, and with that, the higher temperature difference causes more water to evaporate, thereby enhancing the explosion effect. In addition, mechanical effects by the passage through the ball valve and impingement against the inner surface of the blow tank might play a role [25]. A Δ*p* of around 20 bar is necessary to reach the maximum improvement in digestibility, and increasing Δ*p* further gives no more benefit. Analogously, the effect of the explosion on decreasing particle size levels off at a Δ*p* of 20 bar as can be seen from the analysis of the weight-average particle size (Fig. 4a), suggesting that the explosion increases digestibility mainly by the particle size reduction of the biomass.

The required explosion pressure difference for reaching the maximum digestibility or particle size reduction is also dependent on the pretreatment severity. It was, for example, observed that a Δ*p* of 10 or 18 bar in the low-severity pretreatments at 184 and 210 °C (log *R*_0_ = 3.2 and 3.9) did not have any effect on cellulose digestibility, while those pressure differences did, indeed, affect digestibility at the higher severity of 5.0. It can be concluded that a certain pretreatment severity is necessary to soften up the biomass structure so that the explosion can develop its effect. No major influences of Δ*p* on the pH of the pretreatment liquor, the biomass composition or sugars dissolved in the pretreatment liquor could be noticed (results not shown).

The findings on the influence of the pressure difference in explosion on enzymatic digestibility can be compared with the earlier described steam explosion pretreatment study of Brownell et al. [25]. Aspen wood chips were treated at a similar severity of 4.7 (*T* = 240 °C, *t* = 3 min) and a similar pressure of 33 bar. Reduction of steam pressure to 7 bar shortly before the explosion gave a product still almost entirely in chip form, in contrast to the biomass exploded at 33 bar. As opposed to our study, where the reduction of Δ*p* from 30 to 7.5 or 5 bar caused a distinct reduction in digestibility (compare Fig. 4b), it did not have an effect on biomass digestibility in the study of Brownell et al. [25]. The different behavior may be caused by the different types of biomass that were used. Hardwood like aspen generally needs a much lower pretreatment severity than softwood to become digestible, so that the enhancing effect of the explosion may be unnecessary. The different equipment used might also influence the results, though in both studies, the explosion did lead to a visible defibration of the wood chips.

### How does the explosion enhance cellulose digestibility?

As described above, similar trends in decreasing the biomass particle size and in enhancing its enzymatic digestibility were observed, suggesting that these effects are strongly correlated. The influence of biomass particle size on digestibility is not straightforward, and cofactors such as the pretreatment play a role as well [37]. Particle size reduction provides a higher surface area of the substrate and might also open up new or different ways for enzymes to reach the cellulose (e.g. enter pores that were inaccessible before). It is, however, conceivable that the explosion may also alter the micro-/nanostructure of the biomass and exert an influence on enzymatic hydrolysis in that way.

**Fig. 5:**
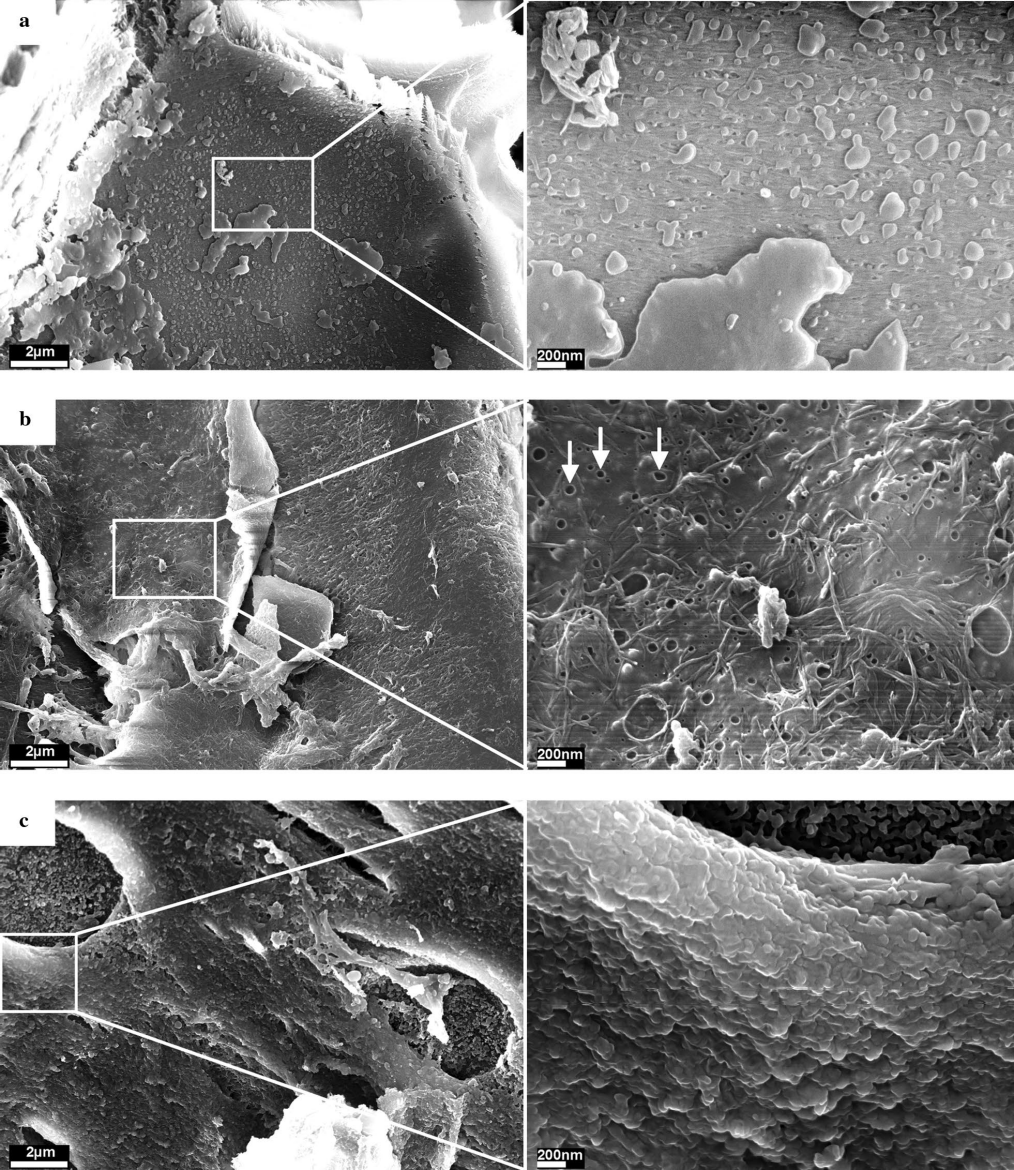
SEM images of pretreated spruce wood chips. **a** After steam pretreatment without explosion. **b** After steam pretreatment with explosion. Cavities on the biomass surface are highlighted exemplarily. **c** After steam pretreatment without explosion and enzymatically hydrolyzed. Pretreatment conditions: log *R*_0_ = 5.0 (*T* = 235 °C, *t* = 10 min); hydrolysis conditions: 1 %w/w cellulose, 60 FPU g^−1^ cellulose

SEM analysis does, indeed, show major differences in the micro- and nanostructure of the distinctly pretreated biomass. Figure 5a shows an example of the biomass surface after a pretreatment without explosion. The surface is covered with many condensed-like structures or droplets, varying in size from several µm to less than 20 nm. The presence of coalesced-like structures is particularly visible in the corresponding lignin residues after enzymatic hydrolysis (Fig. 5c). It has been reported that in autohydrolysis and acidic pretreatments at temperatures above the lignin glass transition temperature, lignin can coalesce and migrate into the bulk liquid phase. Upon cooling, lignin redeposition on the biomass surface can occur in the form of droplets which may have a negative impact on cellulose hydrolysis [38–40].

Studying the surface of the biomass pretreated with explosion shows a very different surface structure (Fig. 5b). No condensed droplet-like structures are visible anymore. It, therefore, seems possible that in the pretreatments without explosion, such structures may have formed upon cooling—when releasing the pressure within about 20 s—and may not be able to precipitate on the biomass during the short time of the explosion. The exploded biomass surface also shows many holes ranging from about 100 to less than 10 nm in diameter (see magnification Fig. 5b), which were not observed in non-exploded samples. Those holes might form when liquid water vaporizes during the explosion and penetrates the biomass surface in the form of steam jets. The biomass surface also shows fibers with a diameter of around 30 nm, which corresponds to the size of cellulose macrofibrils in wood [41]. Those fibers appear to have been exposed and disarranged by the explosion.

**Fig. 6:**
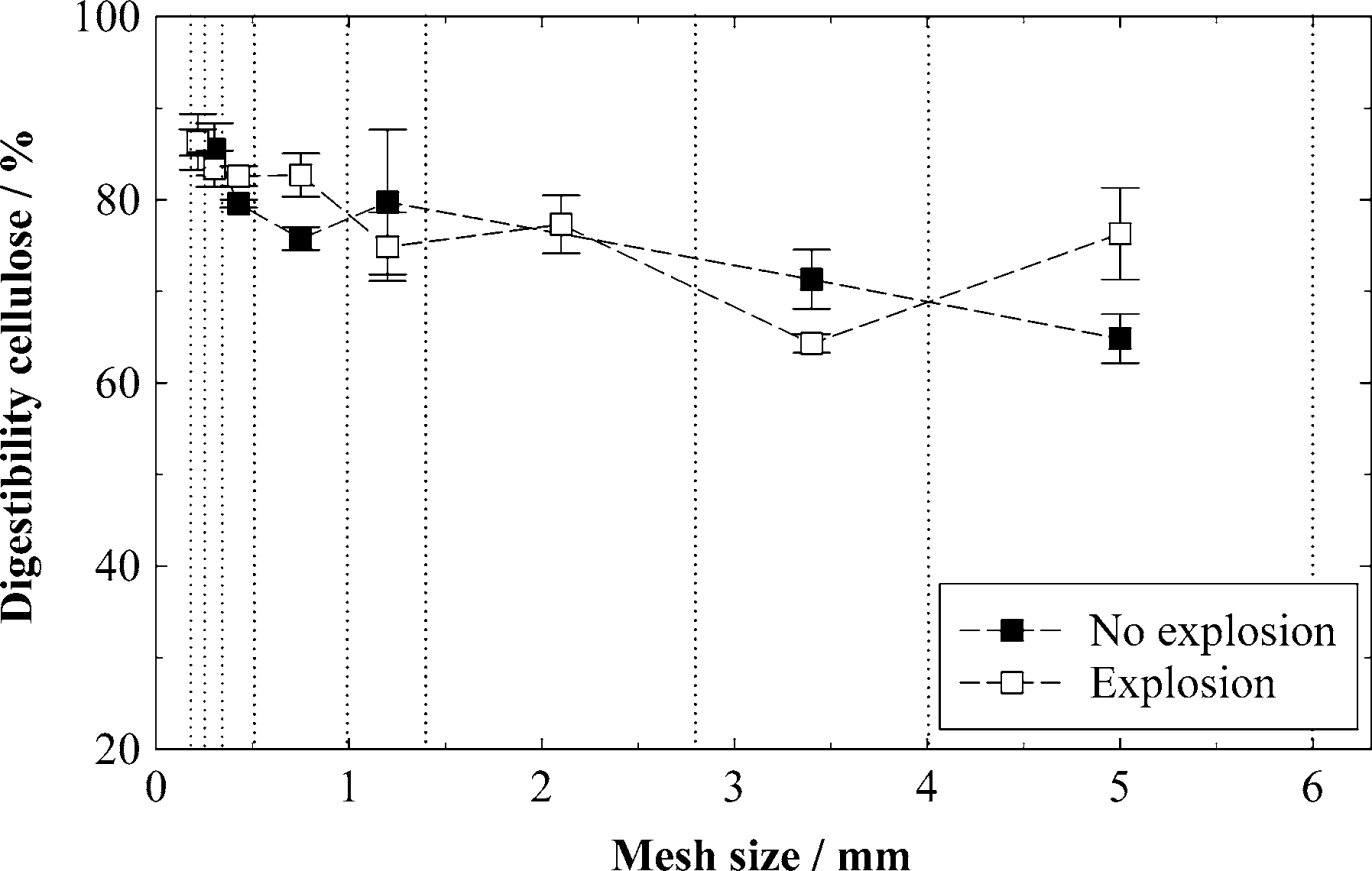
Cellulose digestibility in the enzymatic hydrolysis of fractions of similar particle size isolated from spruce wood chips pretreated with and without explosion. Cellulose conversion to glucose is expressed as % of pretreated biomass content. Fractions were isolated by sieving, the sieve mesh sizes are represented by the dotted vertical lines. Pretreatment conditions: log *R*_0_ = 5.0 (*T* = 235 °C, *t* = 10 min); hydrolysis conditions: 1 %w/w cellulose, 60 FPU g^−1^ cellulose

To estimate the influence of those structural modifications by the explosion on enzymatic hydrolysis, a separate experiment was carried out. Fractions of similar particle size were isolated by sieving from exploded and non-exploded biomass and then enzymatically hydrolyzed. In that way, the influence of the particle size reduction by the explosion on hydrolysis is eliminated. Biomass was taken from experiments where the explosion had led to a high improvement of 61 % relatively compared to the pretreatment without explosion and an almost complete cellulose digestibility (log *R*_0_ = 5.0, Δ*p* = 30 bar; compare Fig. 3a). In this experiment with isolated particle size fractions, it was found that digestibility decreases with increasing particle size (Fig. 6) due to the effects described earlier. However, no enhanced digestibility of the exploded biomass can be observed anymore. This suggests that the explosion influences digestibility mostly via the reduction of the biomass particle size, even though major differences were observed in the biomass surface structure.

**Fig. 7:**
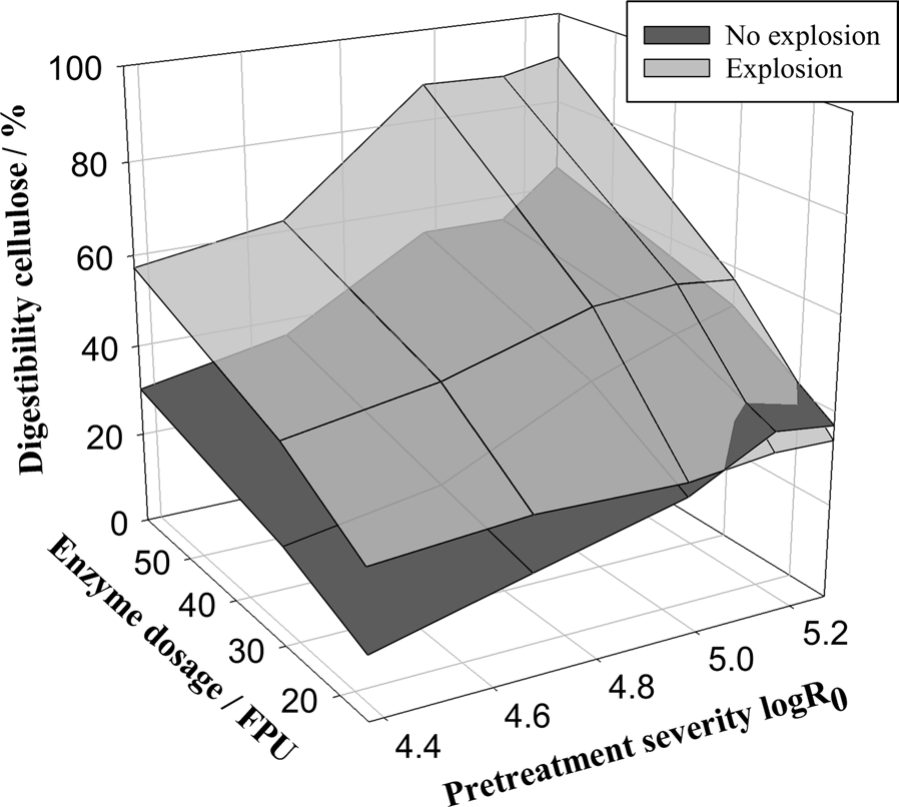
Influence of pretreatment severity and enzyme dosage on glucose yield in enzymatic hydrolysis, expressed as % of pretreated biomass content. Shown are results for spruce wood chips pretreated with and without explosion. Pretreatment conditions: *T* = 235 °C, *t* = 2.5–20 min, Δ*p* explosion = 30 bar; hydrolysis conditions: 1 %w/w cellulose, 15/30/60 FPU g^−1^ cellulose

In a study of the effect of different enzyme dosages in hydrolysis, increasing enzyme dosages can particularly increase glucose yields of the exploded biomass compared to the non-exploded biomass (Fig. 7). This suggests that the explosion effect is, for the most part, based on the enhancement of cellulose accessibility. When hydrolyzing the biomass with lower enzyme dosages of 15 FPU g^−1^ cellulose, even similar hydrolysis yields are reached. Yields at low enzyme dosages are often limited by enzyme deactivation on the pretreated biomass [42], indicating that both biomass samples exert similar levels of enzyme deactivation.

## Conclusions

The explosion in the steam explosion pretreatment of spruce wood chips can greatly enhance its enzymatic cellulose digestibility. The enhancing effect seems to be essentially dominated by the reduction of particle size caused by the explosion. The pretreatment must be carried out both with a sufficient severity to soften up the lignocellulose structure and a sufficient pressure difference in the explosion, so that it can fully develop its effect in defibrating the biomass and decreasing its particle size. If the enhancing effect of the explosion is thoroughly exploited, even very recalcitrant biomass like softwood can be made enzymatically digestible. Though the high cellulose conversions of around 90 % required relatively high enzyme dosages of 60 FPU g^−1^ cellulose, they are still exceptional for a pretreatment process not using any chemicals.

The impact of the explosion on the digestibility of other lignocellulosic feedstock will likely be different. Studying the explosion effect also on biomass like e.g. wheat straw, bagasse or grass crops should give more insights in the enhancing effect of the explosion and also provide valuable information for the pretreatment implementation in biorefinery concepts using steam explosion.

## Additional files

10.1186/s13068-016-0567-1 **Figure S1.** Cumulative particle-size distribution of raw biomass and biomass pretreated with and without explosion. **Figure S2**. Carbohydrate and lignin contents of spruce wood chips after steam pretreatments at different severities with and without explosion. **Figure S3**. pH of pretreatment liquor after pretreatments at different severities with and without explosion.

## Abbreviations

AIL: acid-insoluble lignin
ASL: acid-soluble lignin
FPU: filter paper units
HMF: 5-hydroxymethylfurfural
HPLC: high-performance liquid chromatography
IAP: Industrieanlagen Planungsgesellschaft
NREL: National Renewable Energy Laboratory
SEM: scanning electron microscopy
