# Supplementary material for: Steam explosion pretreatment of softwood: the effect of the explosive decompression on enzymatic digestibility
Source: Biotechnol Biofuels. 2016 Jul 22;9:152. doi: 10.1186/s13068-016-0567-1 (PMC4957380; doi:10.1186/s13068-016-0567-1)
Supplement: Supplementary file 1 — 10.1186/s13068-016-0567-1 Figure S1. Cumulative particle-size distribution of raw biomass and biomass pretreated with and without explosion. Figure S2. Carbohydrate and lignin contents of spruce wood chips after steam pretreatments at different severities with and without explosion. Figure S3. pH of pretreatment liquor after pretreatments at different severities with and without explosion. [file 13068_2016_567_MOESM1_ESM.pdf]

Supporting information for:

Steam explosion pretreatment of softwood - the effect of the explosive decompression on enzymatic digestibility

Thomas Pielhop,* Janick Amgarten, Philipp Rudolf von Rohr and Michael H. Studer*

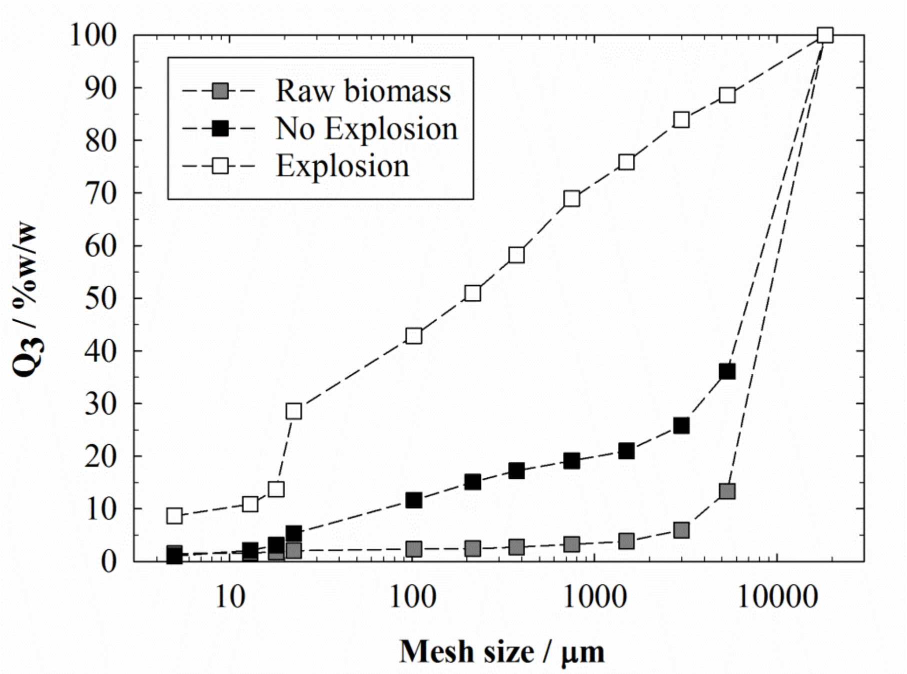

Fig. S1 Cumulative particle-size distribution of raw biomass and biomass pretreated with and without explosion. Pretreatment conditions: logR_0=4.7 (T=235 °C, t=5 min), Δp explosion=30 bar.

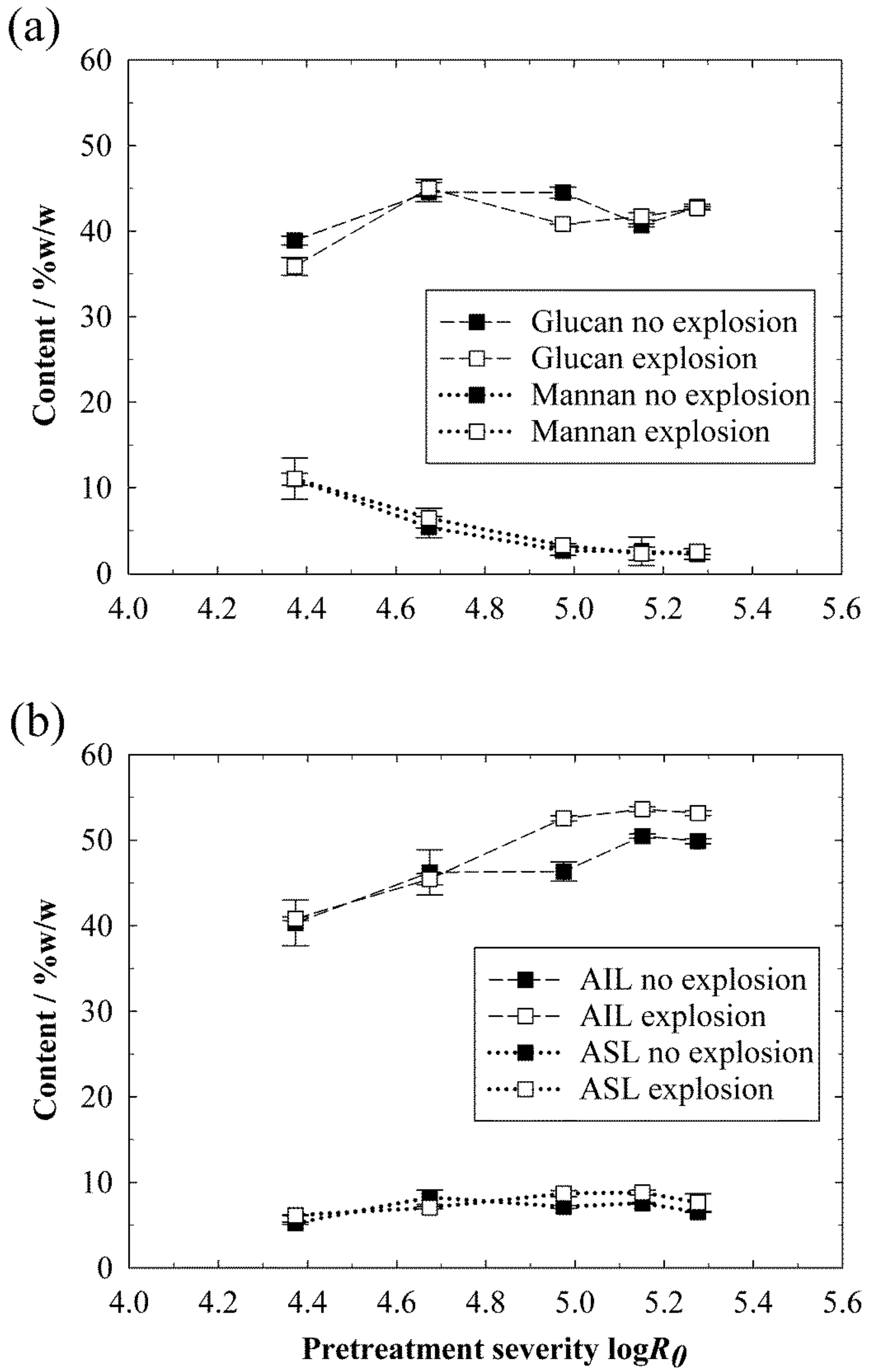

Fig. S2 Carbohydrate (a) and lignin (b) contents of spruce wood chips after steam pretreatments at different severities with and without explosion. AIL: acid insoluble lignin, ASL: acid soluble lignin. Pretreatment conditions: T=235 °C, t=2.5-20 min, Δp explosion=30 bar.

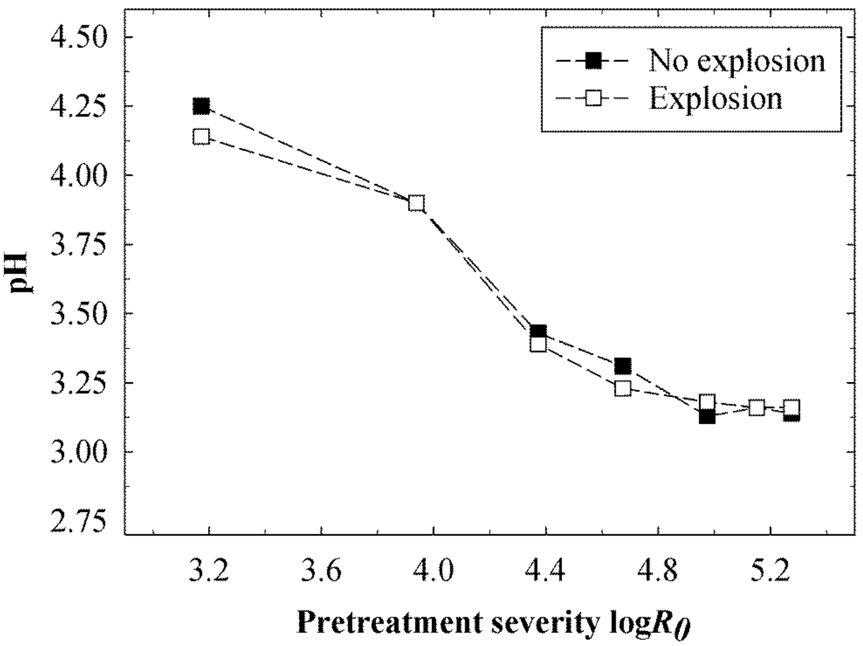

Fig. S3 pH of pretreatment liquor after pretreatments at different severities with and without explosion. Pretreatment conditions: T=184 °C, t=5 min (logR_0=3.2); T=210 °C, t=5 min (logR_0=3.9); T=235 °C, t=2.5-20 min (logR_0=4.4-5.3).
